# Genetic Diversity of *Fusarium oxysporum* f. sp. *cubense* Causing Panama Wilt of Banana in the Philippines

**DOI:** 10.3390/pathogens9010032

**Published:** 2019-12-28

**Authors:** Kristle Grace I. Aguilar-Hawod, Fe M. de la Cueva, Christian Joseph R. Cumagun

**Affiliations:** 1 Institute of Weed Science, Entomology and Plant Pathology, College of Agriculture and Food Science, University of the Philippines Los Baños, Los Baños 4031, Laguna, Philippines; kristle.hawod@stk-ag.com; 2 Institute of Plant Breeding, College of Agriculture and Food Science, University of the Philippines Los Baños, Los Baños 4031, Laguna, Philippines; fmdcueva@yahoo.com; 3 Molecular Phytopathology and Mycotoxin Research, University of Göttingen, Grisebachstrasse, 637077 Göttingen, Germany

**Keywords:** *Fusarium oxysporum* pv. *cubense*, genetic diversity, Luzon, Panama Wilt, Tropical Race 4

## Abstract

Panama wilt, caused by *Fusarium oxysporum* f. sp. *cubense* (Foc) is considered one of the most devastating banana diseases in recorded history. The disease threatens the banana industry due to Tropical Race 4 (TR4) infecting the Cavendish cultivar. Forty-two of the 45 representative isolates from Luzon were pathogenic, based on leaf symptom index and vascular discoloration rating. Accurate, fast and reliable identification are pre-requisites for effective management considering there are yet no proven effective chemicals to control the disease, thus the confirmation by a PCR-based diagnostic tool is essential. Using race-specific primers, FocTr4-F/FocTr4-R and Foc-1/Foc-2, the absence of TR4 in Luzon has been confirmed, however, the occurrence of Race 4 has been reported, which should also be taken in consideration as the latter can also cause severe damage under favorable conditions. Furthermore, to examine genetic diversity of Foc in bananas, 55 of the 164 isolates collected from Regions I, II, III, IV and Cordillera Administrative Region (CAR) were analyzed by fingerprinting techniques using M13, ERIC and REP primers. Twenty-two reference isolates from Mindanao were also analyzed using the same primers. Foc isolates were differentiated into two clades at 25% similarity level, classifying all Mindanao isolates to clade A. Consistently high genetic variation was obtained from Luzon isolates using M13, an arbitrarily primed fingerprinting technique and repetitive elements, REP and ERIC-PCR, while low genetic variation was obtained from Mindanao isolates. ERIC-PCR was the most informative and predictive fingerprinting method as the TR4 isolates from Mindanao were grouped together. No grouping of Foc isolates was observed with respect to geographical origin, except isolates from Mindanao. In addition, grouping of Foc4 is also regardless of host variety in all analyses conducted. Overall, high genetic variability was recorded in Foc Philippine population for the three primers used, which might render host resistance vulnerable.

## 1. Introduction

Banana is second in world fruit production after oranges. It is a vital source of income, employment and export revenues for exporting countries, which are mostly developing countries in Latin America, Southeast Asia and Africa [1]. It is also the fifth most important agricultural crop in world trade [2]. In the Philippines, banana is one of the country’s top export crops, thus considered the most important fruit crop in the country in terms of volume of production and export earnings [3]. The Philippines ranked fifth among the top producing countries of banana, next to India, Ecuador, Brazil and China, respectively. In terms of exports, it ranked third after India and Cost Rica [4]. The total volume of production in 2012 reached 9.2 M metric tons, of which 4.7 M metric tons are Cavendish bananas for export, mainly grown in Mindanao [5].

Several diseases hinder global production, which include Sigatoka or black leaf streak disease caused by *Mycosphaerella fijiensis* and Panama disease or Fusarium wilt caused by *Fusarium oxysporum* f. sp. *cubense* (Foc) [6,7]. Fusarium wilt of banana caused by the soil-borne fungus *Fusarium oxysporum* Schlechtend: Fr. f. sp. *cubense* (E.F. Smith) Snyder & Hansen—Foc, was first reported in Australia in 1874, and as early as 1890 caused an epidemic in Panama, Thus it became known as the Panama disease [7,8]. The disease reached such epidemic proportions that it was considered one of the most destructive plant diseases in recorded history. Panama disease is now found in all banana-producing regions, except the islands in the South Pacific, the Mediterranean, Melanesia, and Somalia [9,10,11]. The presence of resting spores, also known as chlamydospores, make it possible for the pathogen to survive successfully without its host for as long as 30 years, thus susceptible varieties cannot be planted in infested soils for decades [7,12]. In effect, Gros Michel, the previously favored variety for world trade, was wiped out by Fusarium wilt in the middle of the last century forcing the trade to shift to resistant cultivars of the Cavendish subgroup (AAA). The Cavendish cultivar solved the problem for a while; however, in the 1990s, it became susceptible to a virulent strain of Panama wilt, named Race 4 found in Asia. Though in Taiwan, the disease was recorded as early as 1967, the destructiveness of the disease in banana plantations in Indonesia and Malaysia was noted in the early 1990s [12]. To differentiate isolates infecting Cavendish bananas in the tropics and subtropics, Race 4 is further subdivided in Tropical Race 4 (TR4) and Subtropical Race 4 (ST4), respectively [13,14]. While ST4 pathogenicity is enhanced by abiotic stress, TR4 is pathogenic under tropical and subtropical conditions and therefore deemed more aggressive [11,15].

Unlike Sigatoka, which is also a major constraint of production in banana plantains, Fusarium wilt cannot be controlled by fungicides and therefore continues to be a major threat to banana production. Being the country’s biggest export-earning horticultural crop, losses in the banana industry brought about by the destructive Panama disease will likely negatively affect the national economy. In addition, as the disease infects local banana varieties like Latundan, Lakatan and Senorita, small-scale local banana producers will also be affected. Quarantine measures are in effect to control the spread of the disease from the Cavendish plantains in Mindanao to other parts of the country. Thus, a fast and accurate diagnostic procedure is of immense importance to effectively implement these quarantine measures.

There are only a few studies on genetic diversity of Foc in the Philippines [16,17]. Knowledge of the diversity of the pathogen population in specific geographical locations will not only help select Foc-resistant banana cultivars, but may possibly determine the duration of resistance [18]. Most importantly, it will help in the development of appropriate disease management strategies, particularly setting up quarantine measures to prevent spread to new locations, especially in the absence of effective control. Molecular markers have proven effective and are more accurate than phenotypic characterization. Arbitrarily primed PCR technique, such as Random Amplified Polymorphic DNA (RAPD). Used in combination with amplification of the repetitive elements in the genome can be used to characterize diversity of closely related species. The M13 primer has been widely used for fingerprinting a wide variety of filamentous fungi discriminating closely related isolates of a single species, becoming a ‘universal probe’ [19]. Short oligonucleotides such as Enterobacterial Repetitive Intergenic Consensus (ERIC)—PCR, Repetitive Extragenic Palindromic (REP) and BOX Elements initially obtained from prokaryotes, amplify highly conserved inter-repeat sequences in the genome and showed discriminative power to differentiate closely related fungal species [20,21,22].

The objectives of this study are: (1) to test the pathogenicity of representative isolates collected from different parts of Luzon; (2) to determine the occurrence of Tropical Race 4 using race-specific primers from different areas of Luzon, and (3) to examine the genetic diversity of Foc isolates from Luzon and Mindanao using M13, ERIC, REP and BOX primers.

## 2. Materials and Methods

### 2.1. Survey and Collection of Foc

The presence of Fusarium wilt in bananas was surveyed in representative provinces from Regions I, II, III, IV and Cordillera Administrative Region (CAR). Different varieties (Lakatan, Latundan and Saba) were examined for typical wilting symptoms starting from the leaf margin of older leaves extending to the midrib. Pseudostems were then observed for splitting, and an initial cut on a small portion was done to examine discoloration. Samples were collected as pseudostem strands, labeled and placed in separate containers.

### 2.2. Morphological Identification and Maintenance of Foc Cultures

Samples were washed with running water and air dried before processing. Small sections (2 mm × 3 mm) from advancing regions of discoloured pseudostem strands were obtained. These tissues were surface-sterilized using 10% sodium hypochlorite for 5 min and washed in three changes of sterile distilled water and grown in Potato Dextrose Agar (PDA). After 5–7 days incubation at room temperature, mycelial growth from the seeded tissues typical of a *Fusarium*, which are usually white, violet and magenta in color were transferred to PDA slants for purification. Isolated colonies were examined under light microscope for the appearance of macro and microconidia. Upon confirmation, cultures were maintained in Spezieller Nahrstoffarmer Agar (SNA) [23] to avoid degeneration in nutrient-rich media such as PDA. Each isolate was single-spored to ensure purity of cultures [24]. This method was done by pouring spore suspension in dry water agar (WA) plates, and its excess was shaken off immediately. Inoculated plates were incubated in the dark for 18–20 h at an inclined position (30–40°), and examined under dissecting microscope. A single germinated conidium was removed on a small square of agar using a flattened transfer needle and transferred in carnation leaf agar (CLA) to induce sporulation. Colonies that are formed after 5–10 days of incubation were transferred to SNA and PDA slants for further analyses.

Cultural and morphological characteristics were described using the techniques of Nelson et al. [25] and Leslie and Summerell [26]. Data were taken from 12-day old culture of Foc isolate in PDA. Colony color, diameter, hyphal growth, presence of sporodochia were recorded 10 days after incubation. The presence of macro and microconidia and chlamydospores were observed under light microscope.

Aside from periodic transfer of isolates for maintenance, pure culture preservation was prepared by placing sterilized filter paper discs per pure culture of an isolate in SNA slants using forceps. Filter paper discs were grown for at least one week and transferred in to Eppendorf tubes for long term storage.

### 2.3. Pathogenicity Tests

Random representative isolates from different regions of Luzon were selected for a pathogenicity test described by [27] with some modifications. Foc isolates representing each region were inoculated on 6-week-old tissue cultured from Latundan varieties, chosen for its susceptibility to different recognized races of Foc. Inoculum was prepared by transferring 7-day old isolates in sterilized corn meal-sand media. After 10 days of incubation, inoculation on plants was performed by pouring colonized media of each isolate in pots containing the test plants. This is a modification from the original method which described transferring of test plants to pots originally filled with media containing the inoculum. Furthermore, root dipping step, prior to inoculation with corn meal-sand media was also omitted. There were four replicates per isolate representing different areas of collection. During acclimatization and after inoculation, plants were maintained in the greenhouse. Data taken included aggressiveness, which was characterized by the number of days to first symptom appearance; disease severity based on external symptoms following leaf symptom index and vascular discoloration rating scale developed by International Network for the Improvement of Banana and Plantains (INIBAP) [28] with some modifications. Aggressiveness’ parameters were both taken during destructive sampling at 45 days after inoculation (DAI), instead of weekly monitoring, where dead plants are only the ones examined internally, while those that survived were examined at harvest as indicated in the original protocol. Rating scale used are only odd numbers at 1, 3, 5, 7, 9 and 11, instead of the original scale from Carlier with 1, 2, 3, 4, 5 and 6, respectively. The descriptions are as follows: (1) corm completely clean, no vascular discoloration, (3) isolated points of discoloration on vascular tissue (5), discoloration of up to 1/3 of vascular tissue, (7) discoloration of between 1/3 and 2/3 of vascular tissue, (9) discoloration of greater than 2/3 of vascular tissue, (11) total discoloration of vascular tissue.

### 2.4. DNA Extraction and Quantification

Single-spore cultures of each isolate were used for DNA extraction using the CTAB method [29] with some modifications. Isolates were grown in Potato Dextrose Broth (PDB) for 5–7 days without shaking. Mycelia were harvested through the use of sterile filter paper and/or forceps and dried using sterilized paper towel. Mycelia were then freeze-dried and ground to a fine powder in liquid nitrogen using sterilized mortar and pestle. One ml CTAB extraction buffer was added and mixed thoroughly. The resulting solution was then transferred to 1.5 mL Eppendorf tubes, followed by incubation at 55 °C for 1 h. DNA purification was done through addition of phenol-chloroform-isoamyl (PCI), centrifuged for 10 min at 15,000 rpm where supernatant containing DNA was obtained and placed in a fresh Eppendorf tubes. The next step was precipitation with isopropanol at −20 °C for 1 h, followed by centrifugation for 15 min at 13,000 rpm. DNA pellets were washed with 70% ethanol and resuspended in TE buffer. DNA samples were quantified using Nanodrop spectrophotometer and standardize to 50 ng µL^−1^ and 100 ng µL^−1^ with diethylpyrocarbonate (DEPC) water before storing at −20 °C for further molecular analyses.

Foc isolates, tested positive to TR4 under VCG 1213 [30] were provided by the Department of Agriculture in Tagum City as control.

### 2.5. PCR-Based Race Identification

Using race specific primers designed by Dita [27] FocTR4-F)/FocTR4-R for TR4, and Lin [31] for Race 4, DNA was amplified using a thermal cycler (BioRad, Hercules, CA, USA) with the following conditions and some modifications: initial denaturation at 95 °C for 5 min and 30 cycles of denaturation at 95 °C for 1 min, annealing at 60 °C for 1 min and extension at 72 °C for 3 min, followed by an additional extension time for 10 min at 72 °C. FocTR4-F/FocTR4-R and Foc-1/Foc-2 are expected to generate unique amplicon of 463 base pairs (bp) and 242 bp, respectively. Each amplification reaction mixture volume was 15 µL containing 50 ng µL^−1^ DNA, 0.2µM of each primer, 0.2 mM deoxynucleoside triphosphates(dNTPs), 2.5 mM MgCl_2_, 0.6 U *Taq* DNA polymerase, 1X PCR buffer (-MgCl_2_) and DEPC-treated water.

### 2.6. DNA Fingerprinting

To analyze diversity of Foc, arbitrarily primed-PCR using M13 primer and amplification of repetitive sequences in the Foc genome with ERIC, REP and BOX primers were conducted. All amplification procedures using a thermal cycler (BioRad) were done twice to confirm consistency of the method.

### 2.7. Enterobacterial Repetitive Intergenic Consensus (ERIC)-PCR

PCR amplifications were carried out in a 25 μL reaction mixtures using primer pairs ERIC1R (5′-ATG TAA GCT CCT GGG GAT TCA C-3′) and ERIC2 (5′-AAG TAA GTG ACT GGG GTG AGC G-3′) [22]. The reaction mixture consisted of the following components: 1X PCR buffer, 2.5 mM MgCl_2_, 0.2 mM of dNTPs, 0.67 μM of each primer, 1.0 U of Taq polymerase, 50 ng of genomic DNA and DEPC-treated water. PCR amplification conditions were as follows: initial denaturation at 95 °C for 7 min, 35 cycles of denaturation at 95 °C for 1 min, annealing at 48 °C for 1 min and extension at 65 °C for 8 min. Final extension was conducted at 65 °C for 15 min to ensure complete extension.

### 2.8. Repetitive Extragenic Palindromic (REP)—PCR

Using primer set REP1R (5′-IIIICGIICGICATCIGGC-3′) and REP2 (5′-ICGICTTATCIGGCCTAC-3′) in 25 μL reaction mixture with all other PCR ingredients the same as ERIC-PCR, amplification was carried out using the following conditions: Initial denaturation at 95 °C for 7 min, 35 cycles of denaturation at 94 °C for 1 min, annealing at 40 °C for 1 min and extension at 65 °C for 8 min. Final extension was conducted at 65 °C for 15 min to ensure complete extension.

### 2.9. BOX Elements

Reaction was carried out using BOX primer (5′-CTA CGC CAA GGC GAC GCT GAC G-3′) in a 25 μL mixture with the same concentration of PCR ingredients mentioned earlier in ERIC and REP- PCR. Amplification was carried out with the following conditions: initial denaturation at 95 °C for 7 min, 35 cycles of denaturation at 94 °C for 1 min, annealing at 55 °C for 1 min and extension at 65 °C for 8 min. Fifteen minutes final extension was conducted at 65 °C.

### 2.10. RAPD Using M13 Primer

The repeated sequence from the protein II region of M13 phage MR (5′-GAGGGTGGCGGTTCT-3′) one of the two primers, which was previously described for fingerprinting filamentous fungi by [32] was also evaluated. Amplification was performed in a 15-μL reaction composed of 1X PCR Buffer (-MgCl_2_), 1.5 mM MgCl_2_, 0.2 mM dNTP, 1.0 μM of M13 primer and 0.8 U of Taq polymerase and DEPC-treated water with the following conditions: 1 cycle of 94 °C for 4 min, 50 °C for 1 min and 72 °C for 2 min, followed by 40 cycles of denaturation at 94 °C for 1 min annealing at 50 °C for 1 min and extension at 72 °C for 2 min, final extension is carried out at 72 °C for 10 min.

### 2.11. Electrophoresis of PCR Products and Gel Documentation

PCR products were run using an electrophoretic tank (BioRad) containing 0.5X TAE running buffer. For race identification, running conditions include 1.2% agarose at 110 V for approximately 45 min. For all fingerprinting techniques, 1.5% agarose gel was used in 50 V voltage for a total of 3 h running time. Agarose gels were then stained using Gel Red for 25 min, and viewed under Gel Doc (BioRad) equipped with Image Lab Software.

### 2.12. Phenetic Analyses

The genetic relatedness of each isolate was analyzed using the generated banding patterns. A separate data matrix was constructed for each primer by scoring each isolate for the presence or absence of each band. Bands were initially assigned a number in relation to their migration distance within the gel, where highest molecular weight as assigned number one and so on until the band with the lowest molecular weight. It was assumed that the bands with the same molecular weight in different individuals were identical in sequence. To obtain a binary banding data, only strong and reproducible bands were scored, where the presence or absence of each band was determined and designated 1 if present, and 0 if absent. Similarity matrices from binary banding data of each of the three primer combinations were derived with the Similarity for Qualitative Data Program (SIMQUAL) in the Numerical Taxonomy and Multivariate analysis System for personal computer (NTSYS-pc) version 2.1 [33]. Estimates for similarity were based on Jaccards coefficient, where matrices were analyzed and clustered using Unweighted Pair Group Method with Arithmetic averages (UPGMA) as previously described by Bentley et al. [34]. Dendrograms were obtained using the tree option of NTSYS-pc. In order to generate data including all primers used, band matrices from three primer sets were pooled and subjected through the same analyses as mentioned above. Bootstrap analysis of binary data to determine confidence limits was conducted using WINBOOT [35].

## 3. Results and Discussion

### 3.1. Survey and Collection

**Figure 1:**
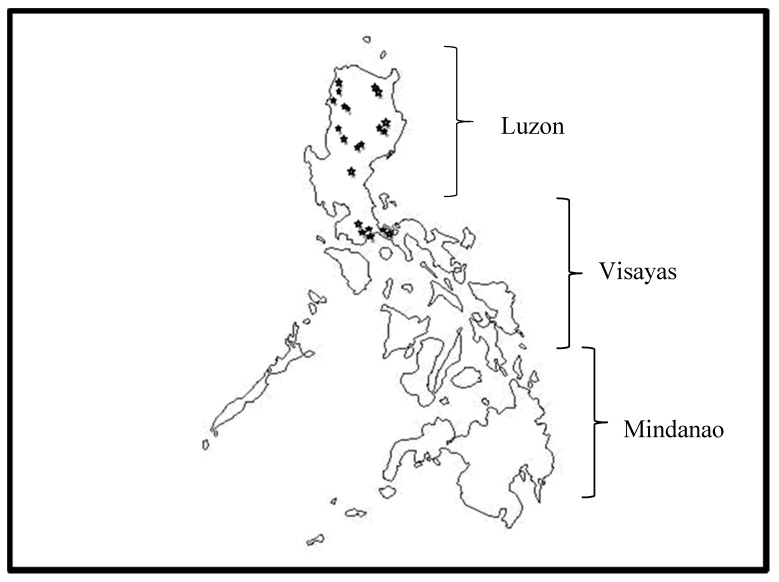
Places of collection in Luzon Philippines.

A total of 164 isolates were collected from 26 areas covering 12 provinces in five regions of Luzon, Philippines (Figure 1 and Table 1). Random sampling was done in areas where bananas exhibited external symptoms characterized by yellowing of the leaf margins of older leaves and/or collapse of leaves at the petiole. Discolorations in the pseudostems were also visible during destructive sampling.

Bananas surveyed in Luzon Philippines, were found mainly as backyard plants used for personal or local consumption (if sold in the local market). Lakatan, Latundan and Saba are the most common varieties grown in the different regions. While Lakatan and Latundan were found in larger farms, which were remote and were not easily accessible, Saba is commonly found alongside the roads or beside houses. Some samples were sent directly to the laboratory, which were collected by the Municipal Agricultural Office in Tiaong, Quezon. Samples collected were immediately isolated in the laboratory. After a 1 to 2 week incubation colonies produced in PDA plate were further purified into monoconidial cultures.

### 3.2. Morphological Characterization

Isolates in PDA varied widely in morphology after 7 days incubation. Color ranged from pale violet, pale orange to dark magenta and some without pigment. Appearance of mycelia varied from flat to aerial, floccose to cottony and circular to irregular form. Some were fast growing, reaching 5 cm in diameter, while others were slow growing reaching only 2 cm after 7 days incubation. Some isolates transformed into pionnotal form characterized by a flat ‘wet’ mycelia colony when frequent subculture is done in PDA. The presence of transposable elements that promote changes in the gene expression might have been the reason behind these phenotypic variation in Foc isolates [36,37].

Microconidia produced by majority of the isolates were oval and kidney shaped, usually single-celled, some with single septation borne from short monophialides and seen as false heads with aerial mycelium presentation. All isolates produced plenty of microconidia, with the exception of 43B2 and 43B3. Macroconidia were usually 3-septated, thin, straight to sickle-shaped with distinct foot cell. Characteristics of chlamydospores which were observed in some isolates greatly varied, with the presence of both smooth and coarse protective wall, which are singly, in pairs or even clusters found in terminal or intercalary. Morphological features are in agreement with the description of Leslie and Summerell [26]. 

Pathogenicity tests were conducted in two batches due to limited ‘clean’ planting materials from the tissue culture laboratories, of National Plant Genetic Resources Laboratory, Institute of Plant Breeding, University of the Philippines Los Baños. Isolates were prepared simultaneously with the potting out of tissue-cultured plantlets in time for inoculation. All isolates fully colonized corn meal-sand media 10 days after incubation.

**Figure 2:**
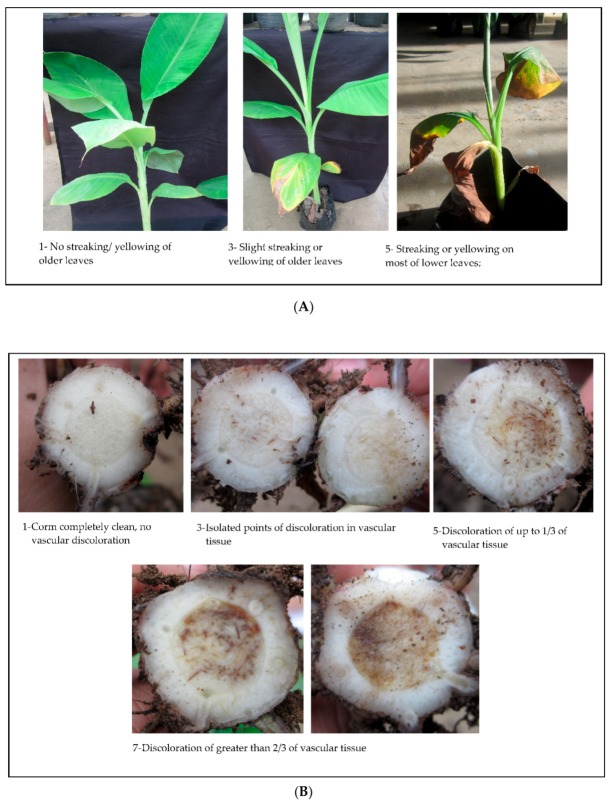
(**A**). Sample representatives of plants with leaf symptoms. Majority of the isolates tested had very mild leaf symptoms in test plants, none of which exhibited symptoms with rating scale of 7 and 9. (**B**). Sample representatives of vascular discoloration with different scale, except rating 9 which wasn’t observed in all isolates tested.

A total of 45 isolates with four replicates were tested representing different places of collection in Luzon (Table 2). There were no pathogenicity tests conducted using Mindanao isolates due to quarantine issues. Variation between the first and second set up include age of test plants which were 6 weeks and 4 weeks old, respectively. Flonicamid, an insecticide for the control of all aphid species was sprayed once at 7 DAI. Based on weekly visual observation, first symptom appearance started at 21 DAI on isolate 24A2a isolated from Lakatan in Mabini, Alicia Isabela, characterized by yellowing of margins in the older leaves. Additional plants manifested wilting symptoms at 28 DAI. Some inoculated plants did not manifest wilting symptoms, but vascular discoloration was evident during destructive sampling at 45 DAI, thus were generally considered not aggressive. Based on the mean of four replicates, majority of the isolates did not manifest leaf symptoms. The highest rating, based on mean of four replicates was 3, characterized by slight streaking or yellowing of lower leaves (Figure 2A). Vascular discoloration rating of isolates 102D1, 24A2a and 116B2.1 were the highest with rating of 5 and 7 characterized by isolated points of discoloration up to 2/3 of the vascular tissue. Mild discolorations up to 1/3 of the vascular tissue were observed in majority of the isolates (Figure 2B). Interestingly, most isolates with high vascular rating also had leaf symptoms. Isolates with highest rating in both parameters for disease severity were generally aggressive based on the days of symptoms appearance after inoculation. Three isolates, 64A2, 74B and 119B1 were considered non- pathogenic, with 0% infection. Considering morphological characterization performed earlier, determination of pathogenic and non-pathogenic elements that might have induced mutation strains of F. *oxysporum* is found to be indistinguishable, consistent with the study of Lievens et al. [38] making diagnosis via morphological characters impossible.

There were no trends as regards to the geographical distribution and host variety where isolates were obtained. Results indicate majority of the isolates being pathogenic; however, the presence of non-pathogenic *F. oxysporum* in the population is possible, which according to Fourie et al. [18] are cosmopolitan and can be found in soils, water and plant residues. Non-pathogenic *F. oxysporum’s* inability to cause disease is attributed to their lack of means to penetrate the vascular tissue or host plants are able to counteract infections [39]. Importance of non-pathogenic strains should also be considered, since it may serve as source of new pathogenic strains [40] due to close genetic relationship between non-pathogenic and pathogenic isolates [41].

Based on the data, it can be concluded that the inoculation procedure used in this study was efficient enough, since most isolates were able to induce infection in Latundan variety leaving the control healthy and symptomless. It can be further deduced that the tested Foc population was not aggressive when compared to other pathogenicity trials conducted, where symptoms usually appear at 7 DAI [27]. However, it should be taken into consideration that there has been no universally acceptable greenhouse inoculation technique for Foc, which is an important bottleneck for characterization, considering that race identification still heavily relies on pathogenicity trials [27,42,43,44]. Following Koch’s postulates, Foc isolates were successfully re-isolated in PDA from rhizomes manifesting discoloration symptoms. Examination under the microscope was done to ensure identity of *Fusarium* isolates obtained.

### 3.3. DNA Extraction of Foc

The CTAB method previously described by other researchers to extract DNA from fungi and related organisms was used successfully on Foc isolates [29,45]. The amount of DNA harvested from 5-day old mycelial cultures in PDB ranged from 20–3000 ng/µL. The use of liquid nitrogen significantly increased the amount of DNA harvested, as compared to extraction using CTAB buffer alone.

### 3.4. Race Identification Using Race-Specific Primers

Given the economic importance of TR4, which is currently threatening the global banana industry, accurate identification will aid in quarantine measures, where effective control has not been established. Race classification of Foc, which is solely based on the capacity to infect differential set of cultivars through pathogenicity tests, are often influenced by different factors such as temperature, age of host, amount of inocula and method of inoculation [46], where results vary from around the world [46,47]. Genetically the same species can be classified into different races based on their capacity to infect differential set of cultivars in different locations, where environmental conditions play a vital role [18]. For instance, field data for TR4 or ST4 should be carefully interpreted, where TR4 is more aggressive than ST4 [15,48], the latter can also cause severe damage in Cavendish cultivars, particularly under abiotic stress such as low temperatures and water logging [15,49,50].

Due to the flaws of pathogenicity testing to determine races, PCR-diagnostic procedure, particularly for Race 4 has been demonstrated several times [18,27,31,51,52]. Specific primer sets were used in this study as a PCR diagnostic tool to detect presence of TR4 and Race 4 in general. FocTr4-F/FocTr4-R primer set was derived from two single nucleotide polymorphism of the intergenic spacer region (IGS) of the nuclear ribosomal operon of Foc specifically designed in TR4 [27]. In South-Central Mindanao in the Philippines, Solpot et al., [17] identified Foc TR4 isolates collected from North Cotabato, South Cotabato, Saranggani, Davao del Sur and General Santos City isolated from Lakatan, Cavendish and Latundan varieties using primers developed by Lin et al. [31].

Isolates from Mindanao produced the expected 463 bp and 243 bp amplicon during gel electrophoresis using primers FocTr4-F/FocTr4-R and Foc-1/Foc-2, respectively. Using primers FocTr4-F/FocTr4-R, results confirmed absence of TR4. Eleven isolates from Luzon gave positive results and produced the expected 243 bp, along with the positive check from Mindanao with primers Foc-1/Foc-2. There were particularly no patterns with regard to the place of collection from Regions 1, II, IV and CAR as well as different varieties including Latundan, Lakatan and Saba from where representative isolates obtained (Table 3).

The results obtained in this study suggest the occurrence of Race 4 in Luzon. Due to the high variation obtained in fingerprinting analyses, our results support the theory of co−evolution where Foc evolved with the edible banana originating in South East Asia. The use of molecular techniques as A more reliable method to detect Race 4 has been widely accepted, and used in the case of reporting the occurrence of TR4 in Indonesia [52] and first report of TR4 outside South East Asia [53]. The race system classification initially helped to discriminate populations, but is already outdated and leads to erroneous conclusions, hampering decision making. For instance, positive results by PCR identification were obtained from isolates in areas where ST4 has not been reported such as in Brazil, Costa Rica, Honduras and the USA [27]. This implies that an isolate can be considered ST4 in areas where it causes symptoms in Cavendish bananas and as Race 1 in areas where it does not affect Cavendish such as in tropical areas in Brazil, Costa Rica and Honduras.

### 3.5. Fingerprinting Analyses

A total of 22 Foc isolates from Mindanao and two TR4 positive checks were analyzed using ERIC−PCR and M13 primer set. Only 58 isolates were subjected to REP analysis. Standardized DNA at 50 ng/µL was used in the different fingerprinting.

### 3.6. ERIC-PCR

**Figure 3:**
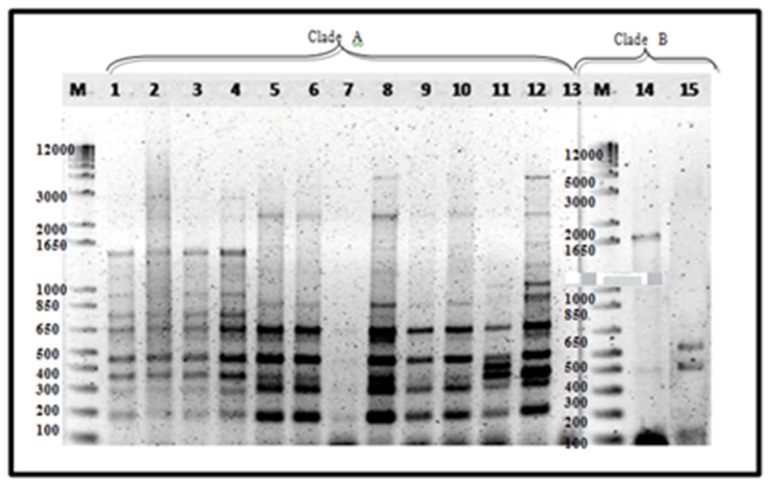
ERIC−PCR amplification of 15 representative Foc isolates. Lane 1, 16B1; 2, 18B3a; 3, 19B2; 4, 24B1; 5, 1Q; 6, 2K; 7, 2G; 8, 3A; 9, 80A; 10, 1213-1; 11, 88A; 12, 89A; 14, 43B2; 15, 43A. Luzon isolates—lanes 1–4, 14–15; Mindanao isolates—lanes 5–12.

ERIC−PCR in Luzon isolates alone amplified a total of 22 banding patterns ranging from 200–5000 bp. Reproducible 3–12 bands of varying intensities were consistent in two analyses conducted. UPGMA cluster analysis showed 19–100% similarity on tested isolates. Two distinct clades were formed, regardless of banana cultivars and geographic distribution. Major bands include 450, 350 and 300 bp for clade A, while 400 and 100 bp are common in clade B (Figure 3).

**Figure 4:**
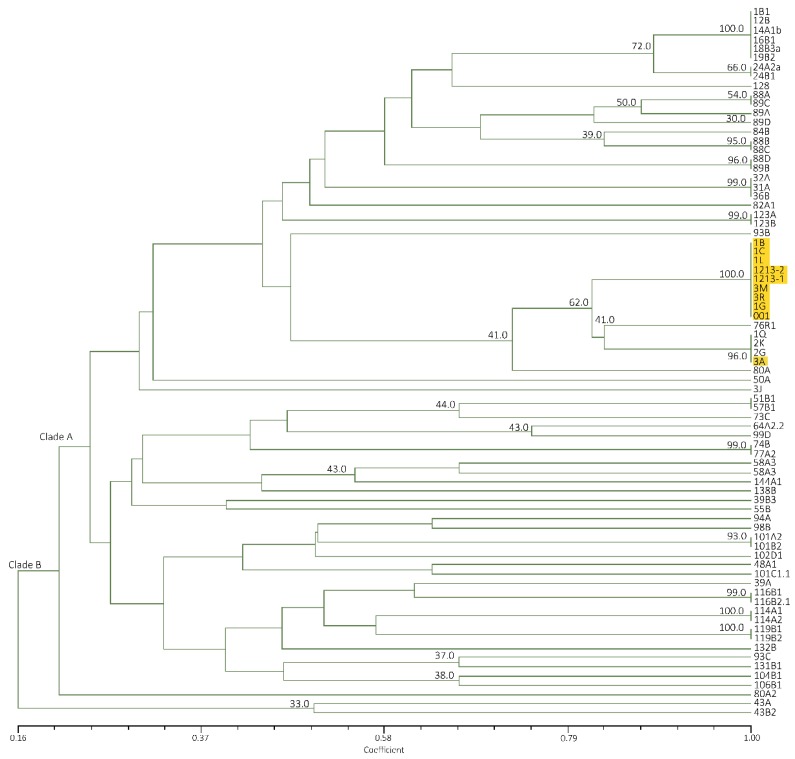
Dendrogram from UPGMA cluster analysis using Jaccard Similarity Coefficient of representative Foc isolates from Luzon and Mindanao based on ERIC-PCR. TR4 isolates are highlighted in yellow.

Mindanao and Luzon isolates generated a similarity value of 16–100% with a total of 22 banding patterns. Two clades were also formed with all Mindanao isolates grouped in clade A having high similarity value at 40–100%, except isolate 3J (Figure 4). The major banding pattern at 650, 450, 300 and 200 bp with an insert at 350 bp is a characteristic feature of Mindanao isolates. Isolates tested positive to Race 4 using primer designed by Lin et al. [31] were categorized in both clades where isolates 24A2a, 31A, 32A, 94A, 101A2, 101B2, 102D1, 104B1,106B1 and 48A1 were under clade A, while only isolate43B2 in clade B. Using ERIC−PCR analyses, Leong et al. [54] likewise categorized isolates from Malaysia and Indonesia into two distinct clades. Furthermore, the suitability of ERIC−PCR was emphasized due to higher variation as compared to PCR−RFLP of ITS+ 5.8S regions [18]. The whole genome is analyzed with ERIC−PCR, as compared to RFLP, which involves specific regions only, giving more information in analyzing closely related species [20,55]. 

All isolates tested successfully generated ERIC banding patterns indicating that repetitive elements could be found in abundance in the FOC genome, consistent with the studies conducted by Leong et al. [56] where 29 banding patterns were observed ranging from 100–3750 and Edel [20] with 19 banding patterns ranging from 100–4000 bp.

### 3.7. RAPD−PCR Using M13 Primer

**Figure 5:**
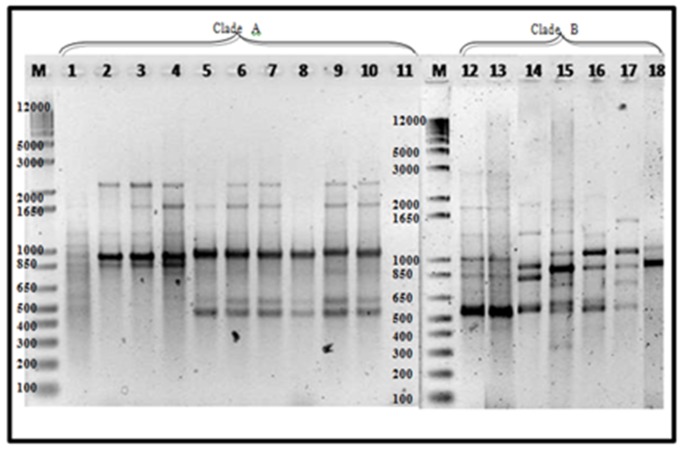
M13 amplification of 17 representative Foc isolates. Lane 1, 1B1; 2, 12B; 3,14A1b; 4, 16B1; 5, 1B; 6, 1C; 7, 2G; 8, 3A; 9, 76R1; 10, 1213-1 (positivecheck); 11, DEPC water; 12, 51B1; 13, 57B1; 14, 43B2; 15, 50A; 16, 74B; 17,77A2; 18, 48A1. Mindanao isolates—lanes 5–10; Luzon isolates-lanes 1–4, 12–18.

The use of M13 primer is considered as RAPD-PCR, since no preliminary information on the target sequence is required. Foc being a haploid asexual pathogen can be effectively analyzed using arbitrary primer techniques such as RAPD and DNA amplification fingerprinting (DAF) [42]. As enumerated by Bentley [57], additional advantages of using RAPD-PCR to study genetic variation include the following: (1) it does not involve the use of cloning, sequencing or radioactively labelled probes, (2) primers can be designed for various levels of differentiation, (3) it is applicable to large numbers of isolates and (4) it analyses variation at more than one locus. Using M13 primer, eighteen 18 banding patterns were observed ranging from 2800 bp to 100 bp (Figure 5). 

**Figure 6:**
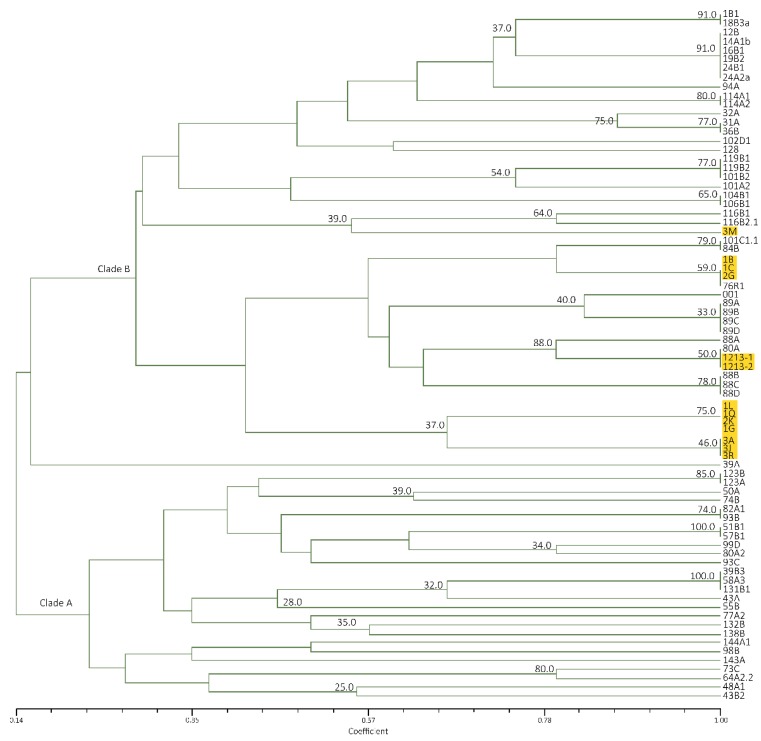
Dendrogram generated from UPGMA cluster analysis using Jaccard Similarity Coefficient of representative Foc isolates from Luzon and Mindanao based on M13 analyses. TR4 isolates are highlighted in yellow.

UPGMA cluster analysis on Luzon isolates revealed a similarity of 18–100% forming two clades. With Mindanao isolates, total of 22 banding patterns of the same range as the latter were observed forming two clades as well with similarity value of 14–100% (Figure 6). Consistent in clade A is the presence of 900 bp major band, while 1000 and 850 bp bands are consistent with majority of isolates belonging to clade B. All Mindanao isolates were grouped together in clade A, consistent with ERIC−PCR analyses where a distinct feature includes the presence of a 450 bp very distinct band and 500 bp faint band. Considering ten isolates that were positive to Race 4 using Foc/Foc2 primers, clade A includes 24A2a, 31A, 32A, 94A, 101A2, 101B2, 102D1 and 104B1, while clade B include isolates 43B2 and 48A1.

### 3.8. REP-PCR

**Figure 7:**
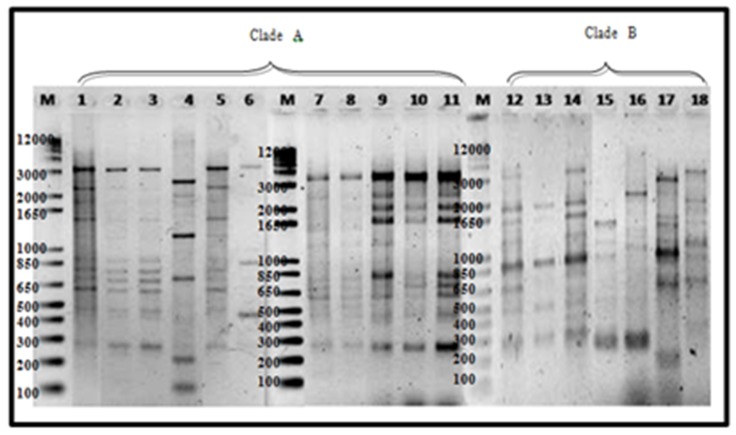
REP amplification of 18 representative Foc isolates: Lane 1, 1G; 2, 2G;3, 3A; 4, 1L; 5, 3R; 6, 001A; 7, DEPC water; 8, 80A; 9, 1213-1 (positive check); 10, 88B; 11, 88C; 12, 88D; 12, 1B1; 13, 12B; 14, 14A1b; 15,43A; 16, 43B2; 17, 51B1; 18, 57B1. Mindanao isolates—Lanes 1 to 12, Luzon isolates—Lanes 12–18. The ladder size is shown in number of base pairs.

Not all isolates generated reproducible bands for REP primer set, thus from the 77 isolates analyzed for M13 and ERIC, 58 isolates, 40 from Luzon and 18 from Mindanao including two positive checks were analyzed using REP−PCR. A total of 22 banding patterns ranging from 5000 bp to 100 bp were formed with varying intensities (Figure 7).

**Figure 8:**
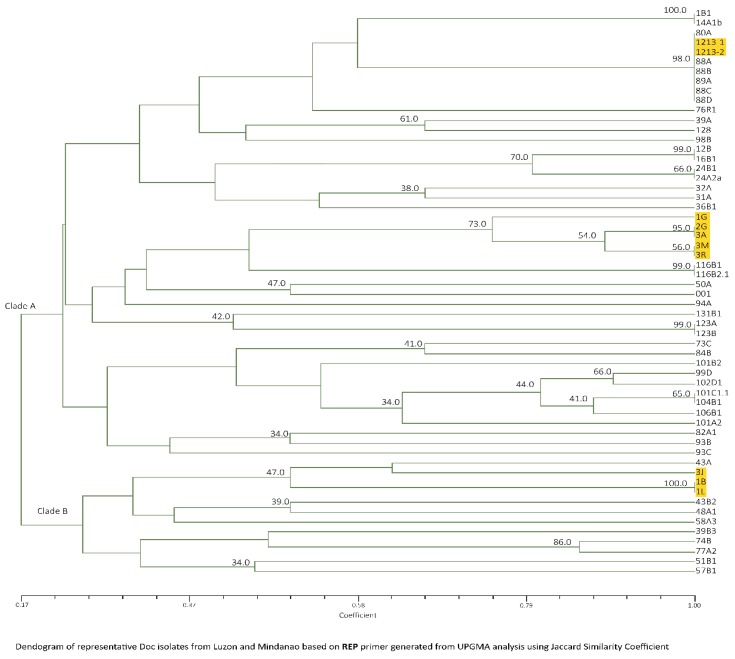
Dendrogram of representative Foc isolates from Luzon and Mindanao based on REP primer generated from UPGMA cluster analysis using Jaccard Similarity Coefficient. TR4 isolates are highlighted in yellow.

Considering Luzon isolates only, UPGMA cluster analysis gave similarity of 14–100% forming two groups, which is in consistent with M13 primer except isolates 116B1 and 116B2.1. With Mindanao isolates, similarity ranged from 17–100% with the same total banding patterns forming two clades as well (Figure 8). Consistent in clade A was the presence of approximately 3000 and 900 bp major bands, while clade B has no consistent major bands, but the presence of 1200 bp was observed with varying intensities. Unique pattern for Mindanao isolates, including positive check is the presence of three bands at 700, 650 and 500 bp under clade A, except 3J, 1B and 1L which was categorized under clade B. Grouping of isolates that were positive to Race 4 is consistent with M13 primer where majority of the isolates belonged to clade A and only isolates 43B2 and 48A1 were under clade B.

### 3.9. BOX Elements

Using BOX primer, very few banding patterns were obtained; some isolates did not even produce a single band. Gradient to optimize PCR conditions as well as variation on the concentration of mixtures had been made, but were not successful. The results suggest presence of BOX elements in Foc, but due to greater sequence conservation, lesser banding patterns were observed (data not shown).

Collectively, results suggest low variability among Mindanao isolates as compared from Luzon collection considering similarity values in all primers used, which can be attributed to intensive monoculture practice in banana plantains. Furthermore, there were particularly no trends with regard to what cultivar were Foc isolated from as well as geographical location. The consistency of having two clades in all analyses is in agreement with the study of Fourie et al. [18], where Philippines isolates included in the study were categorized under clade A, while from Latundan was categorized under clade B. Results indicate high variability among Foc population in the Philippines as compared to genetic variability studies of the pathogen in other countries [58] supporting further the theory of co-evolution by Stover [7], where Foc is believed to have co-evolved with edible bananas and their wild diploid progenitors originating in South East Asia.

Among the three molecular markers used ERIC-PCR was the most informative and predictive, because the TR4 isolates belonged to one group, outperforming RAPD-PCR and REP primers where they were spread apart and at times some TR4 isolates were absent. ERIC-PCR is designed to target repetitive sequences that are highly conserved in the genome. The position of these elements varies from species to species, but is usually 126 bp long, highly conserved at the nucleotide level, and include a central core inverted repeat [59]. In this technique, a band pattern is obtained by amplification of genomic DNA located between ERIC elements or between ERIC elements and other repetitive DNA sequences. Characterization of Foc isolates from Malaysia using ERIC-PCR suggested low variability indicating close genetic relatedness regardless of cultivars and area of collection [54]. We did not conduct combined analysis of the three markers as RAPD−PCR and REP were less informative, which could only weaken the cluster grouping. 

When breeding for resistance, variability studies have been the primary focus of research, particularly for economically important diseases such as Panama disease. Race determination as a phenotypic marker is based on the capacity to infect different banana cultivars under field conditions, where Race 1 infects Gros Michel, Silk, Apple, Lady Finger and Latundan, Race 2 attacks Bluggoe bananas and Race 4 attacks all cultivars susceptible to Foc Races 1 and 2 as well as Cavendish bananas. Race 4 is further classified into ST4 and TR4 to differentiate Foc causing disease to Cavendish in the subtropics and tropics, respectively. However, while ST4 isolates cause disease in Cavendish in the subtropics, mainly when plants are exposed to abiotic stress, TR4 isolates are pathogenic under both tropical and subtropical conditions [15]. The above mentioned race concept complicate identification, and does not capture genetic variation. Therefore, neutral DNA−based technique would be more suitable in analyzing genetic variation within and between Foc populations.

## Figures and Tables

**Table 1:** List of Foc isolates collected from different areas in Luzon, Philippines.

No.	Place of Collection	Variety	Isolate Code
**Region I**			
1	Lubnac, Tagudin, Ilocos Sur	Latundan	IB1, 1B2, 1B3
			12A1, 12A2, 12A3, 12B, 13B1,
			13B2a, 13B2b, 14A, 14A1a,
			14A1b, 14A1c, 14A2, 14A3, 14B1,
2	Bario-an, Tagudin, Iloc Sur	Latundan	14B2, 15A
			16B1, 16B2, 16B3, 18A1, 18A2,
		Tomok	18B1, 18B2, 18B3a, 18B3b
3	Dardarat, Pinili, Ilocos Sur	Unknown	119B1, 119B2
4	Ipet, Sudipen, La Union	Tomok	19A, 19B1a, 19B1b, 19B2, 19B3
			24A, 24A1, 24A2a, 24A2b, 24A2c,
		Lakatan	24A3, 24B1, 24B2
5	Camanggaan, Laoag City	Unknown	122, 123A, 123B, 124A
**Region II**			
6	Fabrica, Lallo, Cagayan	Lakatan	57A, 57B2, 58A2.1, 58A2.2, 58A3,
			60A1, 60A2, 62B1, 62B2
			64A1.1, 64A1.2, 64A2.1, 64A2.2,
		Latundan	67A, 67B1, 69B, 73C
7	Cagoran, lallo Cagayan	Lakatan	55B
8	San Fabian, Echague, Isabela	Latundan	38D, 39A, 39B, 39B1, 39B2, 39B3
9	Poblacion, Cauayan, Isabela	Saba	43A, 43B1, 43B2, 43B3
10	Mabini, Alicia, Isabela	Lakatan	48A1, 48A2, 49B
11	Alinam, Cauayan, Isabela	Lakatan	50A, 50A1, 50A2, 51A, 51B1, 52A
12	Kinakaw, Bagabag, NuevaVizcaya	Saba	29A1, 31A, 31B, 32A, 32B
13	Magsaysay, Bayombong, Nueva Vizcaya	Lakatan	36A1, 36A2, 36B1, 36B2
**Region III**			
14	CLSU, Munoz, Nueva Ecija	Unknown	84B, 85A, 85B
**Region IV**			
15	Tayabas, Quezon	Saba	93A, 93B, 83C, 93D1, 93D2
16	Talisay, Tiaong, Quezon	Latundan	128, 101A2, 101B1.1, 101B1.2,
			101B1.3,101B2.1, 101B2.2, 101B2.3,
			101C1.1, 101C1.2, 101C1.3, 101D,
			101D2.1, 101D2.2, 102B1, 102B2,
			102B3, 102C1, 102C2, 102D1,
17	Tiaong, Quezon	Lakatan	102D2,131A1, 131A2, 131B1, 131B2
18	Bukal Sur, Quezon	Latundan	132A, 132B, 143A1, 142A2, 143B, 144A1,
19	Masalukot II, Candelaria, Quezon	Lakatan	144A2, 144B
20	San Felix, Sto. Tomas, Batangas	Unknown	98B1, 98B2, 99D, 100B1, 100B2
21	Tranca, Bay, Laguna	Latundan	104B1, 106B1
			114A1, 114A2.1, 114A2.2, 114B1,
22	Lawaguin, Nagcarlan, Laguna	Unknown	114B2
23	Sabang, Nagcarlan, Laguna	Saba	116A1, 116B1, 116B2.1, 116B2.2
**CAR**			
24	Luzong, Luba, Abra	Unknown	74B, 75B, 76A, 76B, 77A, 77A2
			79A, 80A1, 80A2, 81B, 82A1,
25	Tobanggao, Poblacion, Abra	Unknown	82A2
26	Tublay, Benguet	Saba	94A, 94B

**Table 2:** Pathogenicity of representative Foc isolates on the Latundan variety of banana expressed in days after first symptoms appearance, leaf symptom index and vascular discoloration rating.

No	Isolate Code	Place of Collection	Variety Where Isolated	Days After First Symptom Appearance	Leaf Symptom Index ^1^	Vascular Discoloration Rating ^2^
1	1B1	Lubnac, Tagudin, Ilocos Sur	Latundan	45	1.00	3.00
2	12A2	Bario-an, Tagudin, Ilocos Sur	Latundan	45	1.00	1.50
3	13B2b	Bario-an, Tagudin, Ilocos Sur	*Latundan*	45	1.00	2.50
4	14A1B	Bario-an, Tagudin, Ilocos Sur	Latundan	35	2.00	3.50
5	16B1	Bario-an, Tagudin, Ilocos Sur	Tomok	45	1.00	2.50
6	18A1	Bario-an, Tagudin, Ilocos Sur	Tomok	45	1.00	3.00
7	19A	Ipet, Sudipen, La Union	Tomok	45	1.00	3.00
8	24A2a	Ipet, Sudipen, La Union	Lakatan	28	3.00	4.50
9	32A	Kinakaw, Bagabag, Nueva Vizcaya	Saba	28	3.00	2.50
10	32B	Kinakaw, Bagabag, Nueva Vizcaya	Saba	28	2.50	1.50
11	36B1	Magsaysay, Bayombong, Nueva Vizcaya	Lakatan	28	1.50	2.50
12	39A	San Fabian, Echague, Isabela	Latundan	28	3.00	3.50
13	39B	San Fabian, Echague, Isabela	Latundan	35	2.50	3.50
14	43A	Poblacion, Cauayan, Isabela	Saba	28	1.50	2.00
15	43B2	Poblacion, Cauayan, Isabela	Saba	35	2.00	2.50
16	48A1	Mabini, Alicia, Isabela	Lakatan	21	2.50	2.50
17	50A	Alinam, Cauayan, Isabela	Lakatan	35	1.50	2.50
18	55B	Cagoran, Lallo, Cagayan	Lakatan	45	1.00	1.50
19	57A	Fabrica, Lallo, Cagayan	Lakatan	45	1.00	1.50
20	57B2	Fabrica, Lallo, Cagayan	Lakatan	45	1.00	2.50
21	58A2.1	Fabrica, Lallo, Cagayan	Lakatan	42	2.00	3.00
22	60A2	Fabrica, Lallo, Cagayan	Lakatan	28	3.00	2.50
23	64A2.1	Fabrica, Lallo, Cagayan	Latundan	0	1.00	1.00
24	67A	Fabrica, Lallo, Cagayan	Latundan	35	1.50	2.50
25	67B1	Fabrica, Lallo, Cagayan	Latundan	42	1.50	2.50
26	73C	Fabrica, Lallo, Cagayan	Latundan	45	1.00	2.00
27	74B	Luzong, Luba, Abra	Unknown	0	1.50	2.50
28	80A1	Tobanggao, Poblacion, Abra	Unknown	35	1.50	2.50
29	81B	Tobanggao, Poblacion, Abra	Unknown	45	1.00	2.00
30	85A	CLSU, Munoz, Nueva Ecija	Unknown	42	2.50	3.00
31	93A	Tayabas, Quezon	Saba	35	3.00	2.50
32	94A	Tublay, Benguet	Saba	35	3.00	2.00
33	99D	San Felix, Sto. Tomas, Batangas	Unknown	35	1.50	3.50
34	101A2	Tiaong, Quezon	Lakatan	28	2.50	1.50
35	101B1.2	Tiaong, Quezon	Lakatan	28	1.50	3.00
36	102C2	Tiaong, Quezon	Lakatan	35	2.00	2.00
37	102D1	Talisay, Tiaong, Quezon	Lakatan	28	3.00	5.00
38	104B1	Tranca, Bay, Laguna	Latundan	45	1.00	4.00
39	114B1	Nagcarlan, Laguna	Unknown	35	1.50	1.50
40	116A1	Nagcarlan, Laguna	Saba	35	2.00	3.00
41	116B2.1	Nagcarlan, Laguna	Saba	35	3.00	4.50
42	119B1	Ilocos Sur	Unknown	0	1.00	1.00
43	131A1	Bukal Sur, Quezon	Latundan	42	1.50	2.50
44	143A2	Masalukot II Candelaria, Quezon	Lakatan	42	2.00	3.00
45	144A1	Masalukot II Candelaria, Quezon	Lakatan	45	1.00	1.50

^1^ Leaf Symptoms Index: 1—no streaking/yellowing of older leaves, plants appear healthy; 3—Slight streaking or yellowing of older leaves; 5—streaking or yellowing on most of lower leaves; 7—extensive streaking or yellowing on all of the leaves; 9—dead/wilted plants. Ratings are mean of four replicates. ^2^ Vascular Discoloration Rating: 1—Corm completely clean, no vascular discoloration; 3—Isolated points of discoloration on vascular tissue; 5—discoloration of up to 1/3 of vascular tissue; 7—discoloration of between 1/3 and 2/3 of vascular tissue; 9—discoloration of greater than 2/3 of vascular tissue; 11—total discoloration of the vascular tissue. Ratings are mean of four replicates.

**Table 3:** PCR-based Race 4 and TR4 identification of Luzon Foc isolates using primers Foc-1/Foc-2 and FocTR4-F/FocTR4-R.

Isolate		Description		PCR-Detection ^1^	
No.	Code	Place of Collection	Variety	FocTR4	Foc1/2 Race 4
1	1B3	Lubnac, Tagudin, Ilocos Sur	Latundan	−	−
2	12B	Bario−an, Tagudin, Ilocos Sur	Latundan	−	−
3	14A1b	Bario−an, Tagudin, Ilocos Sur	Latundan	−	−
4	16B1	Lubnac, Tagudin, Ilocos Sur	Tomok	−	−
5	18B3a	Lubnac, Tagudin, Ilocos Sur	Tomok	−	−
6	19B2	Ipet, Sudipen, La Union	Tomok	−	−
7	24B1	Ipet, Sudipen, La Union	Lakatan	−	−
8	24A2a	Ipet, Sudipen, La Union	Lakatan	−	+
9	31A	Kinakaw, Bagabag, Nueva Vizcaya	Saba	−	+
10	32A	Kinakaw, Bagabag, Nueva Vizcaya	Saba	−	+
11	36A	Magsaysak, Bayombong, Nueva Vizcaya	Lakatan	−	−
12	39A	San Fabian, Echague, Isabela	Latundan	−	−
13	39B3	San Fabian, Echague, Isabela	Latundan	−	−
14	43A	Poblacion, Cauayan, Isabela	Saba	−	−
15	43B2	Poblacion, Cauayan, Isabela	Saba	−	+
16	48A1	Mabini, Alicia, Isabela	Lakatan	−	+
17	50A	Alinam, Cauayan, Isabela	Lakatan	−	−
18	51B1	Alinam, Cauayan, Isabela	Lakatan	−	−
19	55B	Cagoran, Lallo, Cagayan	Lakatan	−	−
20	57B1	Fabrica, Lallo, Cagayan	Lakatan	−	−
21	58A3	Fabrica, Lallo, Cagayan	Lakatan	−	−
22	64A2.2	Fabrica, Lallo, Cagayan	Latundan	−	−
23	73C	Fabrica, Lallo, Cagayan	Latundan	−	−
24	74B	Luzong, Luba, Abra	Unknown	−	−
25	77A2	Luzong, Luba, Abra	Unknown	−	−
26	80A2	Tobanggao, Poblacion, Abra	Unknown	−	−
27	82A1	Tobanggao, Poblacion, Abra	Unknown	−	−
28	84B	CLSU, Munoz, Nueva Ecija	Unknown	−	−
29	93A	Tayabas, Quezon	Saba	−	+
30	93B	Tayabas, Quezon	Saba	−	−
31	93C	Tayabas, Quezon	Saba	−	−
32	94A	Tublay, Benguet	Saba	−	+
33	98B1	San Felix, Sto. Tomas, Batangas	Unknown	−	−
34	99D	San Felix, Sto. Tomas, Batangas	Unknown	−	−
35	101A2	Tiaong, Quezon	Latundan	−	+
36	101B2	Tiaong, Quezon	Latundan	−	+
37	102D1	Tiaong, Quezon	Latundan	−	+
38	101C1.1	Tiaong, Quezon	Latundan	−	−
39	104B1	Tranca, Bay, Laguna	Latundan	−	+
40	106B1	Tranca, Bay, Laguna	Latundan	−	−
41	114A1	Lawaguin, Nagcarlan, Laguna	Unknown	−	−
42	114A2.1	Lawaguin, Nagcarlan, Laguna	Unknown	−	−
43	116B1	Sabang, Nagcarlan, Laguna	Unknown	−	−
44	116B2.1	Sabang, Nagcarlan, Laguna	Unknown	−	−
45	119B1	Dardarat, Pinili, Ilocos Sur	Unknown	−	−
46	119B2	Dardarat, Pinili, Ilocos Sur	Unknown	−	−
47	123A	Camanggaan, Laoag City	Unknown	−	−
48	123B	Camanggaan, Laoag City	Unknown	−	−
49	128	Talisay, Tiaong, Quezon	Latundan	−	−
50	131B1	Bukal Sur, Quezon	Latundan	−	−
51	132B	Bukal Sur, Quezon	Latundan	−	−
52	143A1	Masalukot II, Candelaria, Quezon	Lakatan	−	−
53	144A1	Msalukot II, Candelaria, Quezon	Lakatan	−	−
54	1213=1	Mindanao	Grand Naine	+	+
55	1213=2	Mindanao	Grand Naine	+	+
56	1B	Puyod, Lasang	Grand Naine	+	+
57	1C	Dakudao, Panabo	Lakatan	+	+
58	1G	Bancud, Tagum	GCTCV 219	+	+
59	1I	Puyod, Lasang	GCTCV 219	+	+
60	1Q	GEA, New Corella	Grand Naine	+	+
61	2G	Lapiz, Sto Tomas	Grand Naine	+	+
62	2K	Fabian, Sto. Tomas	Lakatan	+	+
63	3A	Mauro, Calinan	Grand Naine	+	+
64	3J	Lupida, Guiangan, Calinan	Grand Naine	+	+
65	3M	Aweng, Farm, Bunawan	Grand Naine	+	+
66	3R	CFAR BEMCO, Carmen, D. Del Norte	Grand Naine	+	+
67	00-1	Tangub City, San Vicente, Misamis Occidental	Cardava	−	+
68	76R1	Mindanao	Unknown	−	−
69	80A	Mindanao	Grand Naine	−	−
70	88A	Mindanao	Grand Naine	−	−
71	88B	Mindanao	Grand Naine	−	−
72	88C	Mindanao	Grand Naine	−	−
73	88D	Mindanao	Grand Naine	−	−
74	89A	Mindanao	Grand Naine	−	−
75	89B	Mindanao	Grand Naine	−	−
76	89C	Mindanao	Grand Naine	−	−
77	89D	Mindanao	Grand Naine	−	−

^1^ Symbols + indicates positive and − negative specific primer amplification. Numbers 56–77 isolates are from Mindanao analyzed by de la Cueva et al. [6].
